# A Cross-Sectional Retrospective Analysis of the Racial and Geographic Variations in Cataract Surgery

**DOI:** 10.1371/journal.pone.0142459

**Published:** 2015-11-05

**Authors:** Sara Shahbazi, James Studnicki, Charles Wayne Warner-Hillard

**Affiliations:** 1 Department of Health Services Research, College of Health and Human Services, University of North Carolina at Charlotte, Charlotte, North Carolina, United States of America; 2 Department of Public Health Sciences, College of Health and Human Services, University of North Carolina at Charlotte, Charlotte, North Carolina, United States of America; 3 Department of Public Health Sciences, College of Health and Human Services, University of North Carolina at Charlotte, Charlotte, North Carolina, United States of America; The Chinese University of Hong Kong, HONG KONG

## Abstract

**Background:**

Cataract surgery is the most common surgery performed on beneficiaries of Medicare, accounting for more than $3.4 billion in annual expenditures. The purpose of this study is to examine racial and geographic variations in cataract surgery rates and determine the association between the racial composition of the community population and the racial disparity in the likelihood of receiving necessary cataract surgery.

**Methods:**

Using the national prevalence rates from the National Institute of Eye Health and the 2010 Healthcare Cost and Utilization Project—Florida State Ambulatory Surgery Database, we determined the estimated cases of cataract and the actual number of cataract procedures performed, on four race/gender determined groups aged 65 and over in the state of Florida in 2010. The utilization rates and disparity ratios were also calculated for each Florida county. The counties were segmented into groups based on their racial composition. The association between racial composition and disparity ratios in receiving necessary cataract surgery was examined. The Geographic Information System was used to display county-level geospatial relationships.

**Results:**

African-Americans have a lower gender-specific cataract prevalence (African-American male = 0.246, African-American female = 0.392, white male = 0.368, and white female = 0.457), but they are also less likely than whites to receive necessary cataract surgery (utilization rate: African-American male = 7.92%, African-American female = 6.17%, white male = 12.08%, and white female = 10.54%). The statistical results show no overall differences between the disparity ratios and the racial composition of the communities. However, our geospatial analyses revealed a concentration of high racial disparity/high white population counties largely along the West Coast and South Central portion of the state.

**Conclusions:**

There are racial differences in the likelihood of receiving necessary cataract surgery. However, there is no significant statewide association between the racial composition of the community population and the racial disparity in the likelihood of receiving necessary cataract surgery. Geospatial techniques did, however, identify subpopulations of interest which were not otherwise identifiable with typical statistical approaches, nor consistent with their conclusions.

## Introduction

Cataracts are the leading cause of blindness worldwide and the number one cause of poor vision in the U.S. [1]. Cataract is considered as a serious public health issue among the elderly because it decreases quality of life by increasing the risk of injury, affecting the ability to perform daily living activities, thereby reducing patient’s independence and causing depression and social isolation [2]. “Cataracts are extremely common; more than half of Americans ages 65 or over have a cataract, and it is thought that almost everyone will develop one if they live long enough” [3]. In the United States (U.S.), about 300,000–400,000 cases of visually disabling cataracts occur annually [4]. The total number of persons who have cataract is estimated to increase to 30.1 million by 2020 and of these 9.5 million are predicted to undergo cataract surgery [5].

Treatment of cataract involves removal of the clouded natural lens. The opacified lens is usually replaced with an artificial intraocular lens called an Intraocular Lens Implant (IOL) [6]. The surgery is performed as an outpatient procedure, and does not require an overnight stay. Cataract extraction is now one of the most commonly performed outpatient procedures with more than three million such surgeries performed each year [2]. It is estimated that Americans spend $6.8 billion annually on direct medical costs for outpatient, inpatient and prescription drug services related to the treatment of cataract [7]. Patients who undergo cataract surgery usually experience a significant improvement in visual function, satisfaction with their vision, and quality of life [8]. However, there is still unequal access to the cataract treatment for some populations including racial minorities.

Reducing racial disparities in healthcare utilization and outcome has been consistently highlighted as a national priority [9]. For the disease or condition in which treatment choices are either made electively or selected among several alternatives, the existence of the racial disparity may be due in part to systematic differences in physician discretion or patient preferences [10]. However, for cataract, different treatment rates among racial groups largely reflect the racial disparity in receiving the necessary treatment because the selection of treatment alternatives by both patients and physicians is, for most patients, limited to surgery (IOL) [11]. Several previous studies have examined the factors that might influence the receipt of cataract surgery, including: patient age, sex, race, income, environmental (latitude of residence), access to a facility, medical insurance, and access to regular medical care [12–20]. Moreover, a number of population-based longitudinal studies in recent years have characterized lens opacities and their treatment across various racial groups [21–24] and have generally documented lower utilization of cataract surgery in African–American patients. However, very few studies to date have had a sufficiently large and diverse population to comprehensively evaluate racial differences in utilization of cataract surgery.

Geographic variations in healthcare also frequently correlate with race and poverty [25] and geospatial analyses play an increasingly important role in identifying racial disparities and the interventions aim at reducing them [26]. One of the advantages of geospatial analyses relative to traditional analysis methods is that visualization can explore the results of traditional statistical analysis and identify "unusual" spatial patterns, exceptions and outliers, and formulate hypotheses to guide future research [27]. Moreover, it allows policy makers to easily understand and visualize the problems in relation to the resources, and effectively target resources to those communities in need [28]. Using visual and spatial skills, data features including population characteristics (i.e., racial and gender composition) and spatial attributes (i.e., spatial accessibility to healthcare services) “can be perceived before conscious attention, “pre-attentively”, so they become understandable at a glance, and much more rapidly than words” [29]. To our knowledge, geographic variation in racial disparities in receiving cataract surgery has not been previously described.

Furthermore, previous studies have addressed the existence of significant residential clustering by race among different regions of the U.S. [26]. African-Americans and whites are not equally represented in different geographic regions and studies have documented that this uneven distribution of populations contributes to health disparities-particularly for African-Americans, who studies consistently show are most likely to live apart from other racial-ethnic groups [26]. This residential segregation by race has been proven to be significantly correlated with the existence and persistence of health disparities [30, 31]. The effect of race-based residential segregation on inequalities in healthcare utilization has received increasing attention from researchers. It is suggested that racial residential segregation can reinforce racial differences in both access to care and the quality of care [30]. However, there is a no study addressing whether residential segregation is related to the cataract treatment received by African-Americans and whites.

The purposes of the present study are: 1) to determine the statewide cataract surgery rates among non-Hispanic African-American and White older adults, 2) to examine the county-level racial and geographic variations in receiving the necessary cataract surgery among these populations, 3) to assess whether the racial composition of the community population influences the racial disparity in the likelihood of receiving cataract surgery, and 4) to apply geospatial techniques along with traditional statistical methods in the field of health disparity.

## Methods

### Data Source

Using the Agency for Healthcare Research and Quality's (AHRQ) Healthcare Cost and Utilization Project (HCUP)- State Ambulatory Surgery Database (SASD), we analyzed data from Florida 2010, which capture 100 percent of ambulatory procedures performed on the same day in which patients are admitted and released [32]. The SASD is a de-identified and publicly available data [33]. We chose to use Florida data because of its racially and ethnically diverse population and huge concern about sun exposure and risk of age-related cataracts for its residents. In addition, Florida was ranked in the 1st place in the proportion of people aged 65 years and older in the U.S. [34].

### Study Population/Setting

For our primary analysis, we restricted our study population to non-Hispanic persons 65 years or older because of the low prevalence of cataract among younger people. We excluded Miami-Dade because of its disproportionately large population of Hispanics and concerns about threats to the validity of ethnic assignment [35]. Furthermore, using the *Current Procedural Terminology* (CPT) codes, we restricted our cohort to patients who underwent an Extra Capsular Cataract Removal with Insertion of Intraocular Lens Prosthesis (CPT codes 66882–66984).

### Analytic Approach

Using data from the 2010 Florida Atlas, we identified all Non-Hispanic African-American and White, aged 65 and older in the state of Florida. Using the national prevalence rates from the National Institute of Eye Health, we estimated the expected number of persons affected by cataract in the state of Florida in 2010 and for population aged ≥ 65 by multiplying the national prevalence rates of each race/gender group into their total number of older adults. We used 2010 HCUP—SASD in order to identify the actual number of cataract procedures which were performed on four race/gender determined groups (white females, African-American females, white males, and African-American males) for the state overall and each of 66 counties.

The utilization rate also was calculated for each of the 66 Florida counties and also for each age/gender group by dividing the total number of cataract surgeries performed in our study population by the estimated number of cataracts cases. We then calculated the racial disparity ratio of cataract surgery use. The numerator for our rate calculation was cataract surgery utilization rate of white population. The denominator represented cataract surgery utilization rate for African-American patients. A disparity ratio of greater than one indicates a higher white utilization rate; a ratio of less than one indicates a higher African-American cataract surgery utilization.

Next, we divided the 66 counties into groups based on the fraction of their African-American population:

Group1: < 5% African-American [includes 17 counties: (n = 818,561 whites), (n = 30,411 African-Americans)];
Group2: 5-<10% [22 counties: (n = 1,069,636 whites), (n = 84,138 African-Americans)];
Group3: 10<15% [15 counties: (n = 318,504 whites), (n = 44,836 African-Americans)];
Group4: 15-<20% [4 counties: (n = 269,817 whites), (n = 56,896 African-Americans)]; and
Group5: ≥ 20% [8 counties: (n = 201,496 whites), (n = 59,687 African Americans)].

In the next step we examined the cataract surgery utilization rates and disparity ratios in these categories.

#### Statistical Analysis

One-way ANOVA analyses were used to compare mean differences in disparity ratios across racial composition categories.

STATA 12 (College Station, TX) was used for all statistical analyses. *P* ≤ 0.05 was considered statistically significant.

#### Spatial Analysis

Using the Geographic Information System tool we displayed racial differences in the likelihood of receiving necessary cataract surgery. We also displayed the categories of the racial composition on a map in order to produce a visual presentation of the level of spatial segregation by race across the state. In the next step, we added the racial disparity ratios to GIS layers of racial composition groups in order to create a county-level choropleth map of racial disparity in receiving cataract surgery across the racial composition groups.

GIS Mapping ArcView 9.1 (Environmental Systems Research Institute) was used to perform GIS functions.

## Results

### Statistical Results

Table 1 shows detailed information on the population size, state-level national prevalence and the estimated number of older adults affected by cataract, total number of cataract surgeries performed, and cataract surgery utilization rates for individual race/gender groups.

**Table 1:** Estimated Cataract Surgery Rate, by Race and Gender, 65 years and Over, Florida 2010.

	White Female	African-American Female	White Male	African- American Male	Total White	Total African-American
Total Population > = 65	1467101	160284	1210913	115684	2678014	275968
Prevalence Rate*	0.457	0.392	0.368	0.246	—	—
Estimated Cataract Patients	670465	62831	445616	28458	1116081	91290
Cataract procedures performed**	79204	4655	59572	2687	138776	7342
Cataract Surgery Utilization Rate	10.54%	6.17%	12.08%	7.92%	11.15%	6.71%

* Prevalence Rate: Obtained from National Institute of Eye

** Identified in the HCUP-SASD Florida 2010

The cataract surgeries performed among population aged ≥ 65 accounted for 146,118 of the 3,014,809 total ambulatory surgical procedures in 2010 Florida HCUP-SASD. Furthermore, from the Table 1, 94.98% of all discharges were white and 5.02 were African-American. The state-level results show that African-Americans have a lower gender-specific cataract prevalence (African-American male = 0.246, African-American female = 0.392, white male = 0.368, and white female = 0.457), but they are also less likely than whites to receive necessary cataract surgery (utilization rate: African-American male = 7.92%, African-American female = 6.17%, white male = 12.08%, and white female = 10.54%). Table 1 shows females have higher cataract prevalence, but less likely to undergo cataract surgery than males.

**Fig 1:**
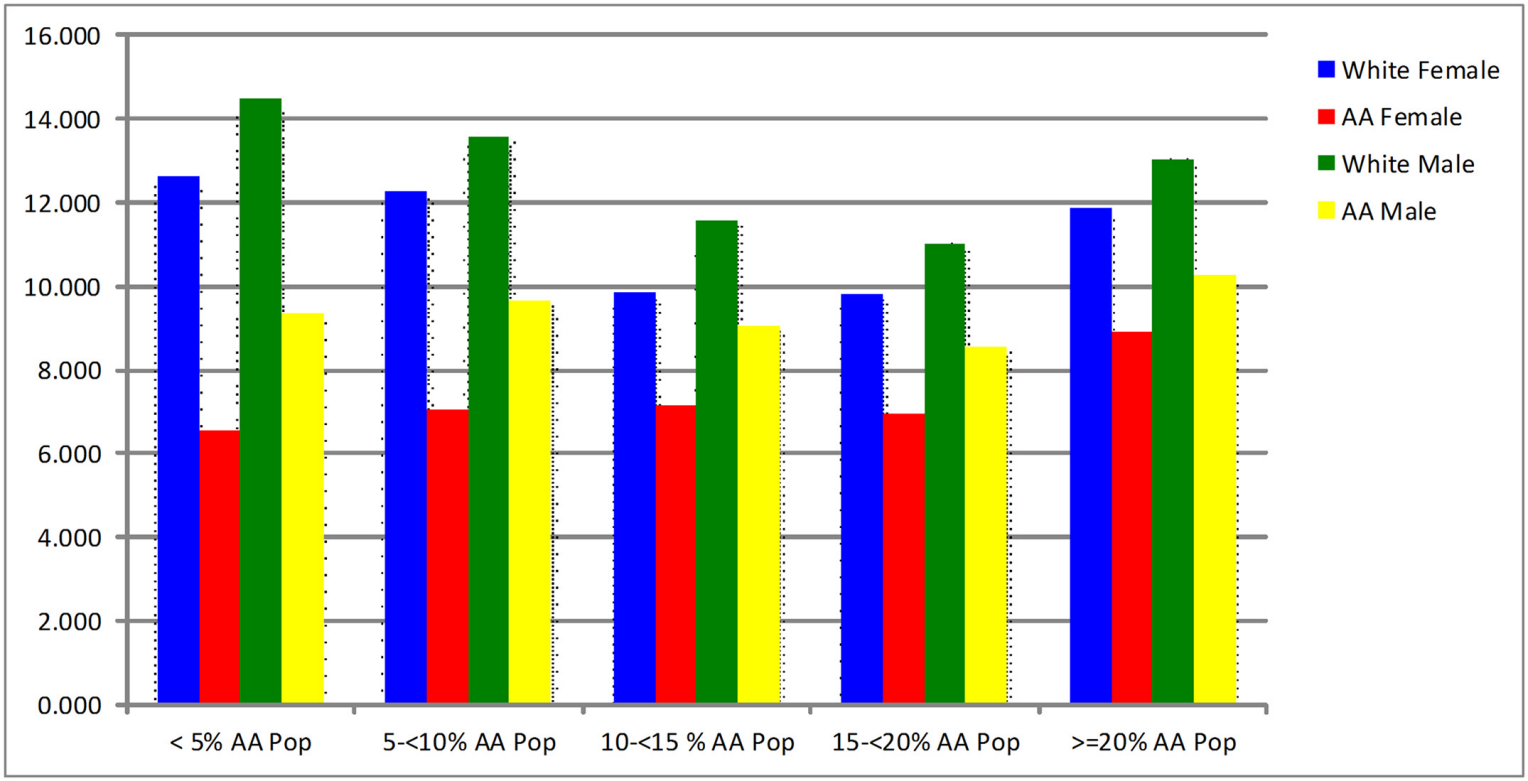
Estimated Cataract Surgery Rates in County Categories based on Percentages of African-American Population in Race/Gender Groups.

In the stratified analysis by racial composition (Fig 1), we found no significant differences in means of disparity ratios for receiving necessary cataract surgery between African-Americans and whites across the racial composition categories, suggesting that utilization of cataract surgery does not change significantly as the African-American population in an area increases. Whites were more likely to receive the necessary cataract surgery regardless of the African-American composition of the community. Our statistical analyses also revealed no statistically significant differences in rate of disparity across the racial composition categories (*F* = 1.51, *P*-value = 0.2103).

### Geospatial Results

**Fig 2:**
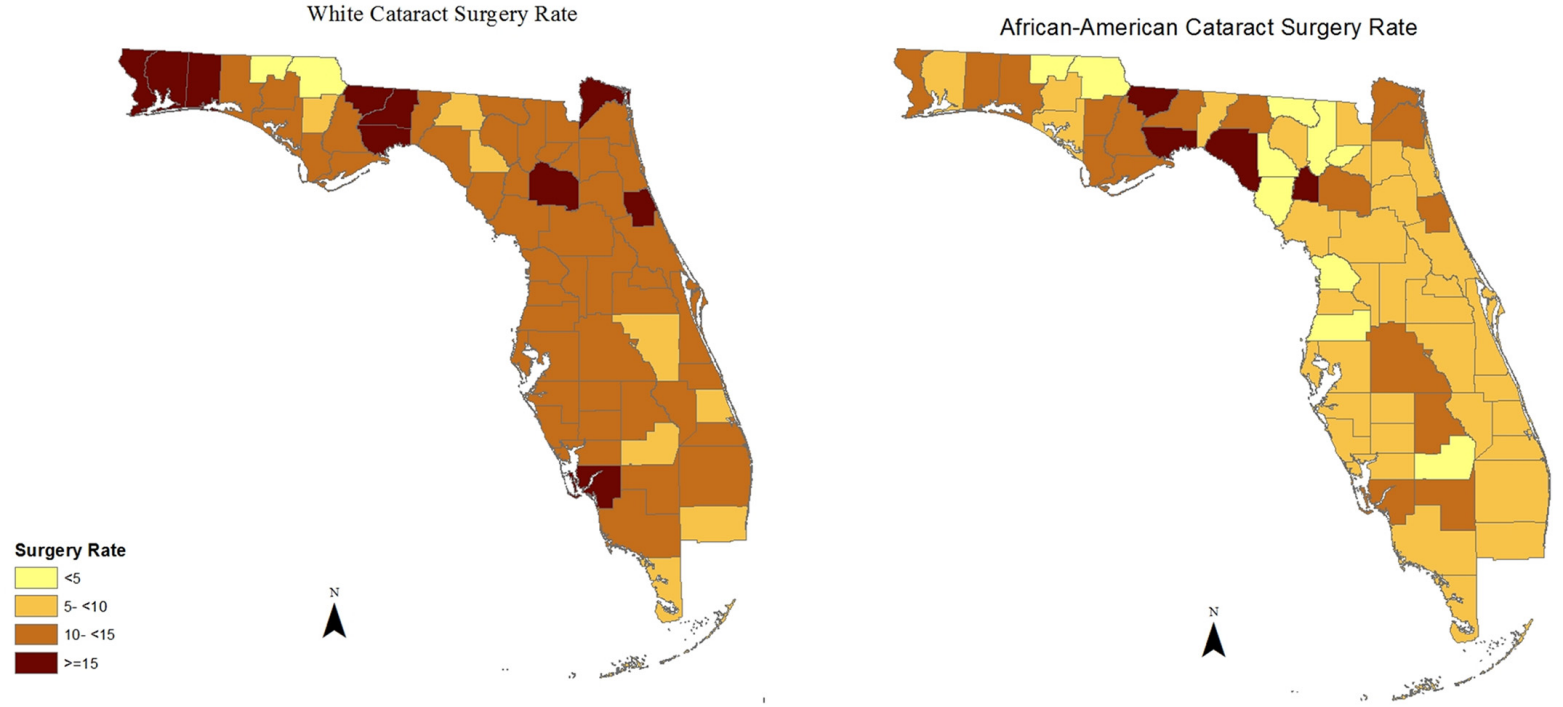
African-American vs. White Cataract Surgery Rate.

**Fig 3:**
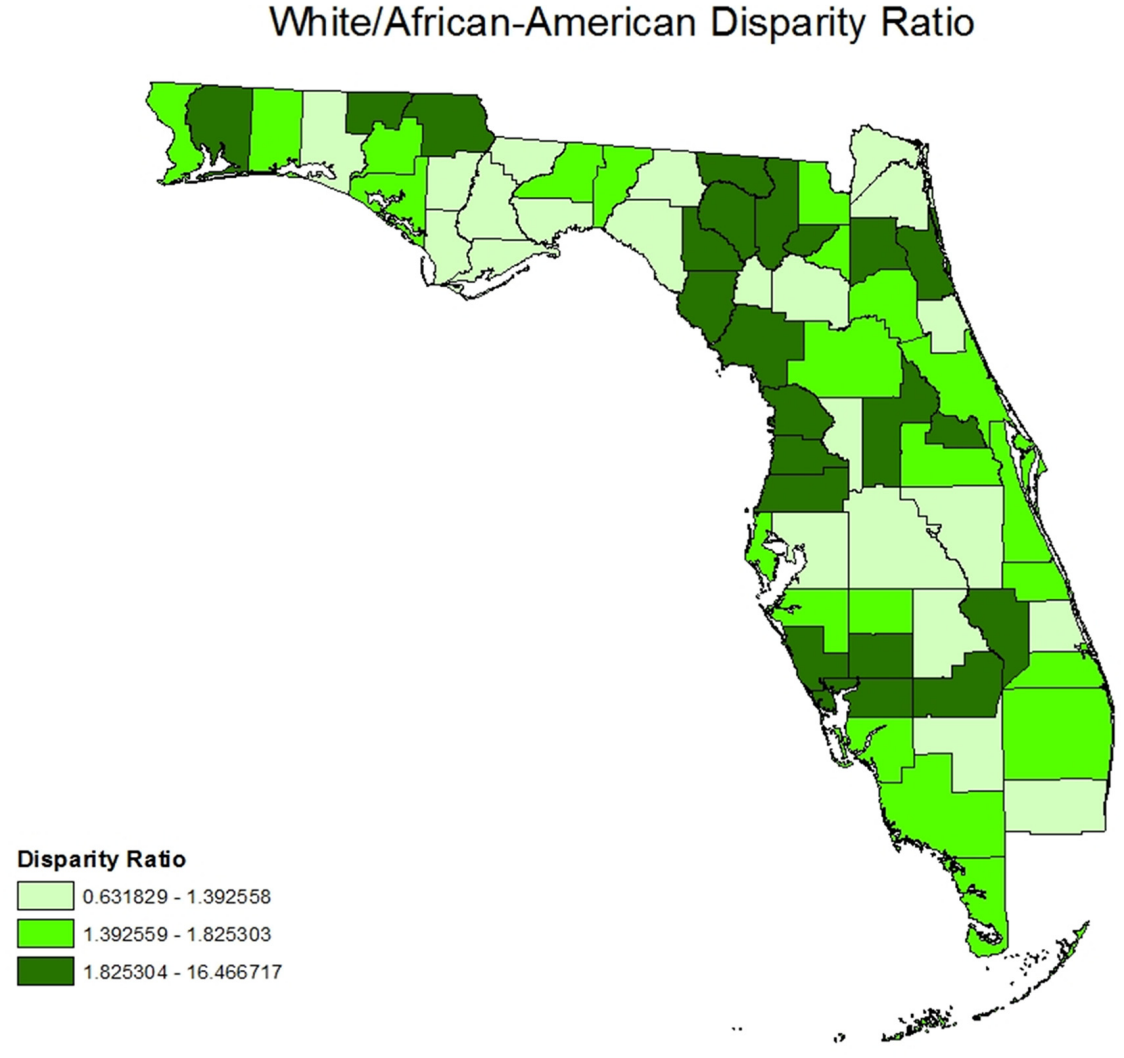
Racial Disparity in Rate of cataract Surgery (Disparity Ratios).

**Fig 4:**
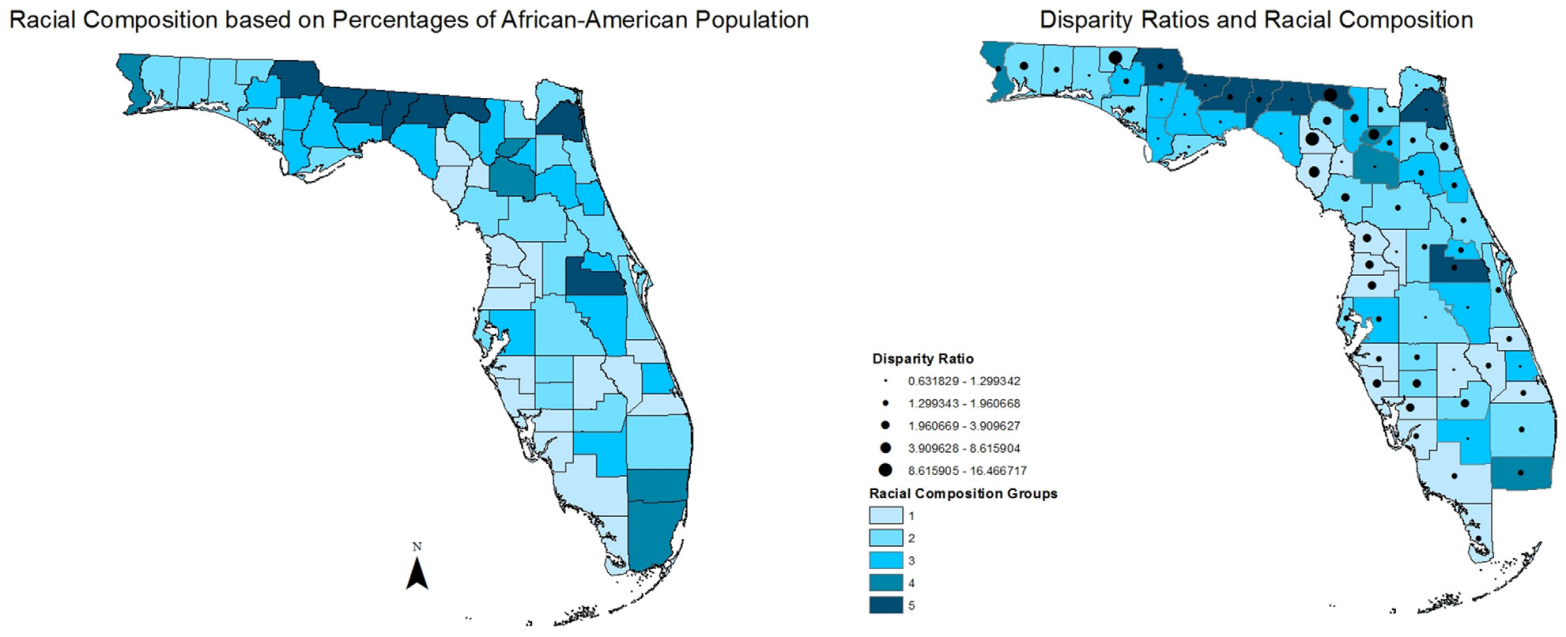
Racial Disparity in Utilization of Cataract Surgery across the Racial Composition Categories.

Our geospatial results also support the existence of racial differences in the likelihood of receiving necessary cataract surgery (whites overall are more likely to have cataract surgery) (Fig 2). Examining the racial disparities in utilization of cataract surgery at county-level, as shown in Fig 3, reveals an unexplained coastal difference with higher racial disparities in a group of counties, most of which are located in an “L shaped configuration” from the Georgia border, South along the Western (Gulf of Mexico) Coast, and then East across the Middle South portion of the state. Moreover, our geospatial results also confirm that there is no statewide association between racial composition and disparity in receiving necessary cataract surgery (Fig 4). As shown in Fig 4, there is no pattern of increased disparity ratios in areas with higher proportion of African-American population compared to racial composition groups with less proportion of African-Americans. However, visual comparison of Figs 2 and 3 suggest that there is a concentration of high racial disparity/high white population counties largely along the West Coast and South Central portion of the state, but this pattern is not apparent consistently within the entire state. It is important to note that the majority of these counties are small, have large older population, belong to racial composition groups with lower percentage of African-Americans, and have lower median household income than the state median (Table 2).

**Table 2:** Characteristics of the Counties with high racial disparity along West Coast and South Central portion of the state.

County name	Total population	Population > = 65	Population > = 65(%)	Racial composition group	Median age	Rank by area*	Median household income
Hamilton	14799	1971	13.32	5	38.1	58	$37613
Suwannee	41551	7994	19.24	2	42.2	36	$36352
Columbia	67531	10519	15.58	3	39.9	25	$38214
Union	15535	1607	10.34	4	39.1	67	$41794
Lafayette	8870	1115	12.57	1	36.2	55	$46445
Dixie	16422	3202	19.50	1	45.4	33	$32312
Levy	40801	7993	19.59	2	45.0	9	$35737
Citrus	141236	45245	32.04	1	54.0	47	$37933
Hernando	172778	44856	25.96	1	47.7	62	$42011
Pasco	464697	97158	20.91	1	43.6	30	$44228
Sarasota	379448	119077	31.38	1	52.5	50	$49388
Desoto	34862	6373	18.28	2	38.1	40	$35979
Charlotte	159978	54856	34.29	1	55.9	34	$45037
Glades	12884	2821	21.90	2	43.1	27	$39429
Okeechobee	39996	6872	17.18	1	38.6	28	$38339

* County rank by land area

State Median Age: 37.2 [36]. State Median Household Income 2010: $47661 [36].

## Discussion

Cataract is the leading cause of visual impairment among the elderly in the U.S. and cataract surgery is the most effective and successful treatment and should be offered to all who can benefit from it. Most previous research show an increased cataract rate among Whites, and also among women as well as a lower utilization of cataract surgical services among African-Americans [21– 24].

Our results are consistent with previous population studies in showing the existence of racial disparities in utilization of cataract surgery. Consistent with previous studies, our study found that non-Hispanic African-Americans have lower cataract prevalence but they are also less likely than whites to receive necessary cataract surgery. Our results revealed high cataract prevalence/ low cataract surgery rate among females compared to males. We also found no overall association between the racial composition of the community population and the racial disparity in the likelihood of receiving necessary cataract surgery, which is inconsistent with previous studies that have generally suggested that an increase in the proportion of African-American or Hispanic population in an area is associated with a decrease in the use of surgical services [37].

As health disparities are often geographically specific, it is particularly important to consider residential segregation with respect to race to understand and address their causal factors. Our findings highlight the advantages of application of geospatial techniques in medical and health services research. The geospatial techniques enabled us to explore an unusual pattern for racial disparity in receiving cataract surgery in an area of the state which was not identifiable in the statistical analyses. We visualized the racial residential segregation of cataract surgery and identified a band of high racial disparity/high white population counties largely along the West Coast and South Central portion of the state. Our Further analyses revealed that the identified band of counties mostly includes small populations and low household income counties with an older population but with a small percentage of African-Americans. Furthermore, because Florida is the fourth most populous U.S. state and has a high level of racial/ethnic diversity, it is a good source of data for studying racial/ethnic variations in the U.S. population.

Despite the strengths of this study, it relied on national race and gender specific data to model prevalence estimates. No statewide assessment of cataract development among the population is available. In addition, our administrative data does not allow a measure of cataract development and severity. It is also worth noting that our study population, people 65 and over, is universally insured under Medicare and thus the influence of income on utilization is likely somewhat mitigated.

Over the coming decades, the total number of Americans ages 65 and older will increase sharply [38]. As a result, an increasing number of older Americans will be living with cataract and disabilities resulting from it, consequently, resources will be required to meet their needs for cataract treatment. Further studies are warranted to help better understand barriers to access, particularly with regard to race, gender, and geographic factors, and to identify the underlying reasons for such disparities.

## Conclusion

Racial and geographic variation in utilization of cataract surgery persists. The geospatial technology is helpful in exploring regional racial disparity patterns which may not be identifiable in traditional statistical analyses. Assessing currently available resources within the geographic areas with higher racial disparity seems necessary to unravel the complexities of racial/ethnic disparities, and begin to identify the target for vulnerable subpopulations interventions.

## Abbreviations

AHRQ: Agency for Healthcare Research and Quality's
SASD: State Ambulatory Surgery Database
GIS: Geographic Information System
CPT: Current Procedural Terminology
IOL: Intraocular Lens Implant
HCUP: Healthcare Cost and Utilization Project
